# Network Pharmacology-Based Antioxidant Effect Study of Zhi-Zi-Da-Huang Decoction for Alcoholic Liver Disease

**DOI:** 10.1155/2015/492470

**Published:** 2015-04-02

**Authors:** Li An, Fang Feng

**Affiliations:** ^1^Department of Pharmaceutical Analysis, China Pharmaceutical University, Nanjing 210009, China; ^2^Key Laboratory of Drug Quality Control and Pharmacovigilance, China Pharmaceutical University, Ministry of Education, Nanjing 210009, China

## Abstract

Zhi-Zi-Da-Huang decoction (ZZDHD), a classic traditional Chinese medicine (TCM) formula, has been used for centuries to treat alcoholic liver disease. Reliable therapeutics of ZZDHD has also been validated in clinical practice. In this study, molecular docking and network analysis were carried out to explore the antioxidative mechanism of ZZDHD as an effective therapeutic approach to treat alcoholic liver disease. Multiple active compounds of ZZDHD were screened based on four key original enzymes (cytochrome P450 2E1, xanthine oxidase, inducible nitric oxide synthase, and cyclooxygenase-2) involved in ethanol-induced oxidative stress damage. A drug-target network was constructed through network pharmacology analysis, which predicted the relationships of active ingredients to the targets. Some results had been verified by the previous experimental pharmacological studies; meanwhile, it was first reported that xanthine oxidase and eriocitrin, neoeriocitrin, isorhoifolin, and poncirin had interactions. The network pharmacology strategy used provided a forceful tool for searching the mechanism of action of TCM formula and novel bioactive ingredients.

## 1. Introduction

Alcoholic liver disease (ALD) is one of the major diseases threatening human health and is also the leading cause of liver-related morbidity and mortality worldwide [1, 2]. Although no single process or underlying mechanism can account for all the effects of alcohol on an organism or even on one specific organ [3], it has been proven that reactive oxygen species (ROS) and oxidative stress play central roles in the early stage of the disease process [4]. In ethanol-induced liver damage, ethanol can increase the generation of reactive oxygen species (ROS) such as O_2_^−•^, H_2_O_2_, and excessive oxygen free radicals disrupt the balance between the oxidation and antioxidation systems, further causing oxidative damage. Moreover, ROS cause the lipid peroxidation of cellular membranes [5], and lipid peroxidation in turn further aggravates ROS generation [6]. Thus, oxidative stress results from excessive oxygen free radical formation and antioxidant depletion due to the ROS generated by ethanol administration [7].

In early-stage alcoholic liver damage, these two enzymes, cytochrome P450 2E1 (CYP2E1) and xanthine oxidase (XO), have been proven to generate ROS in liver as a consequence of alcohol exposure [8, 9]. Ethanol mainly increases the activities of CYP2E1 and XO, which can generate excessive oxygen free radicals and lead to lipid peroxidation, further causing oxidative stress damage. Oxidative stress mainly increases the activities of inducible nitric oxide synthase (iNOS) and cyclooxygenase-2 (COX-2), further aggravating lipid peroxidation and oxidative stress [10, 11]. In ethanol-induced liver damage, four enzymes including CYP2E1, XO, COX-2, and iNOS are the original proteins described as generating ROS or oxidative stress, and they play important roles in the damage due to oxidative stress. Hence, the timely and effective control of high activities of these enzymes, which would eliminate ROS or relieve oxidative stress, is beneficial for the treatment of alcoholic liver injury.

Zhi-Zi-Da-Huang decoction (ZZDHD), a traditional Chinese medicine (TCM) formula, was first described in the book of Jin-Kui-Yao-Lue (*Synopsis of Golden Chamber*). ZZDHD, consisting of four crude herbs:* Gardenia jasminoides* Ellis (Zhi-Zi),* Rheum officinale *Baill (Da-Huang),* Citrus aurantium* L. (Zhi-Shi), and Semen Sojae Preparatum (Dan-Dou-Chi), has been reported to have the ability to treat or alleviate the symptoms of alcoholic jaundice, alcoholic liver disease, and acute hepatitis [12]. Wang et al. [13] reported that ZZDHD had a significant protective effect by reversing biochemical parameters and histopathological changes, and its antioxidant function ameliorated the hepatic injury induced by alcohol. Thirty active components of ZZDHD have also been reported [14]; however, it remains unclear how the active ingredients function as antioxidants.

Network pharmacology, a key technology of system biology, has attracted much attention by researching the molecular mechanisms of TCM formula for complicated diseases [15–17]. Zhang et al. [18] studied an integrative platform of TCM network pharmacology and its application on a herbal formula. Li et al. [19] also determined active compounds and action mechanisms of Ge-Gen-Qin-Lian decoction for treatment of type 2 diabetes by network pharmacology method. Many active chemical compositions of TCM target multiple proteins in the biological network of some disease. Molecular docking is available for modeling interactions between small molecules and proteins. Thus, research of TCM based on network pharmacology, which is a holistic understanding of the molecular mechanisms responsible for the pharmacological effects of herbal medicines [17, 20], is well worth exploration.

In this study, a network pharmacology study of ZZDHD was established through molecular docking and network analysis based on thirty identified active components of ZZDHD and four potential targets including cytochrome P450 2E1 (CYP2E1), xanthine oxidase (XO), inducible nitric oxide synthase (iNOS), and cyclooxygenase-2 (COX-2). The study provides a powerful tool for explaining the antioxidant mechanism of TCM formula and discovering novel bioactive ingredients.

## 2. Materials and Methods 

### 2.1. Potential Targets and Ligand Structures Preparation

Reactive oxygen species are the major prooxidant agents in oxidative stress-induced lipid peroxidation, which results in oxidative damage to various types of cell components including lipids, proteins, and DNA [21–23]. The crystal structures of all candidate targets were retrieved from RCSB Protein Data Bank (http://www.pdb.org/), and relevant proteins including CYP2E1 (PDB ID code 3E6I), XO (PDB ID code 3NRZ), COX-2 (PDB ID code 3PGH), and iNOS (PDB ID code 1M8E) were chosen as potential targets of oxidative-stress damage. All proteins were performed using the CHARMm Force Field with the help of the software package Discovery Studio 2.5 (Accelrys, USA). A total of 30 chemical structures of ZZDHD were identified by HPLC-PDA-ESI-MS/MS in our early laboratory work (see Figure S1, available online at http://dx.doi.org/10.1155/2014/492470), and all the structures of these compounds were optimized by MMFF94 Force Field in DS 2.5.

### 2.2. Molecular Docking and Network Building

Molecular docking was conducted with the LibDock protocol based on the CHARMm Force Field in DS2.5. LibDock is considered to balance speed and accuracy and is based on the matching of polar and apolar binding site features of the protein-ligand complex. In the docking procedure, high quality was set for the docking preference parameter, and the best was set for the conformation method. The other parameters were used as the default. In general, the protein-ligand docking active site is defined by the location of the original ligand. A compound of ZZDHD was considered to be a potentially active ingredient if the LibDockScore of the compound was higher than the original ligand. Conversely, the compound was not considered if it was not shown at the binding site of the protein-ligand complex. The drug-target network was then constructed using Cytoscape 3.0.2 software (http://www.cytoscape.org/) based on the top 10 of the molecular docking rank. In the network, nodes stand for compounds and targets, and edges represent the compound-target interactions.

## 3. Results and Discussion

### 3.1. Network Construction of Molecular Docking-Based Pharmacology

**Figure 1:**
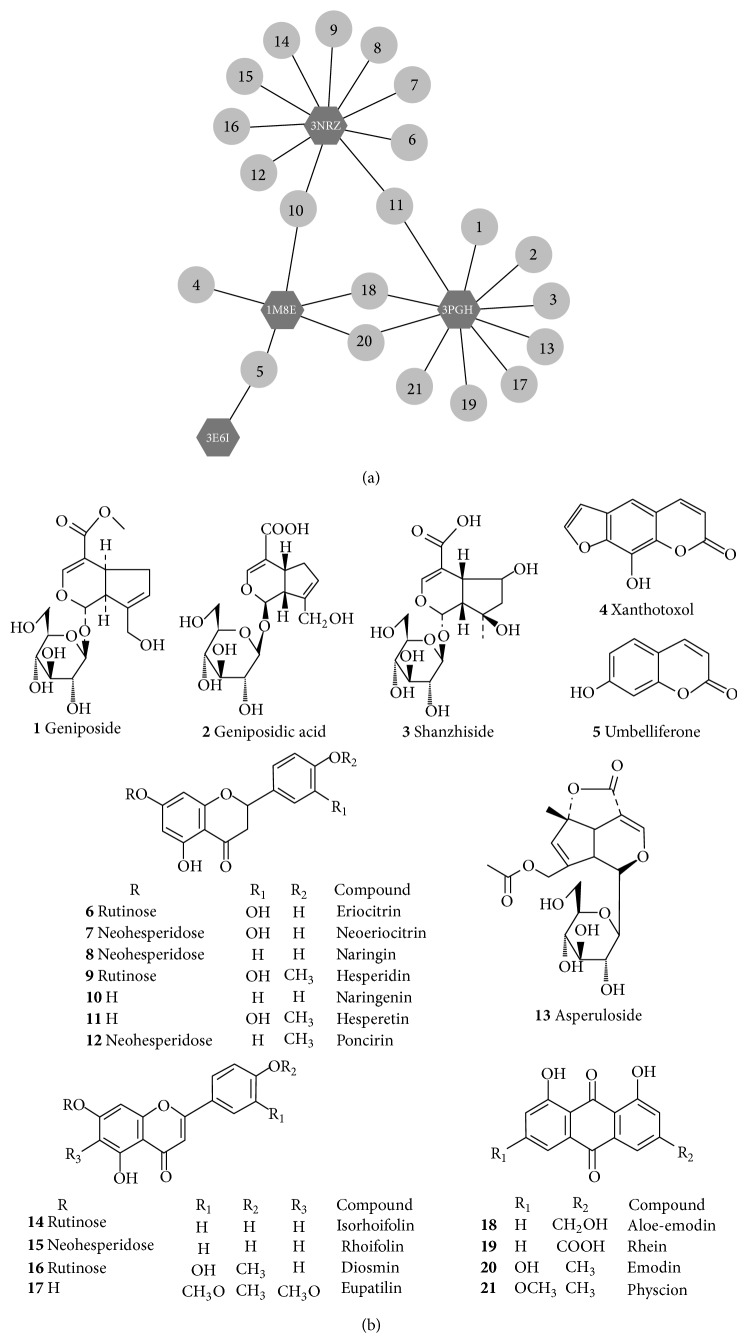
The drug-target network related to antioxidative mechanism of ZZDHD in ALD was shown in (a), CYP2E1 (PDB ID code 3E6I), XO (PDB ID code 3NRZ), COX-2 (PDB ID code 3PGH), and iNOS (PDB ID code 1M8E). (b) displayed the corresponding chemical structures of the 21 active components from ZZDHD.

The active ingredients of ZZDHD were predicted through molecular docking. To further illuminate the relationship between effective compounds and potential targets, a drug-target network was built through network analysis (Figure 1). Multiple active pharmaceutical ingredients of ZZDHD were found to affect different targets.

The network showed the interactions of XO with 10 compounds such as hesperetin and naringenin, COX-2 with 10 compounds such as aloe-emodin and hesperetin, and iNOS with 5 compounds such as aloe-emodin and naringenin; CYP2E1 showed interactions with only umbelliferone. Previous laboratory pharmacological studies have provided much information about the chemical compounds screened and the corresponding targets. Park et al. reported [24] that aloe-emodin dose-dependently inhibited iNOS mRNA expression and nitric oxide (NO) production at 5–40 *μ*M. In addition, the levels of cyclooxygenase-2 (COX-2) mRNA and prostaglandin E2 (PEG_2_) production were suppressed by 40 *μ*M aloe-emodin. This result indicated that aloe-emodin was an effective inhibitor for these two targets. Jayaraman et al. [25] have proved that naringenin decreased the expression of iNOS and COX-2 in the liver of ethanol fed rats. Moreover, Park et al. [26] investigated the inhibiting mechanism of naringenin at the molecular level, finding that naringenin inhibited iNOS and COX-2 mRNA expression and reduced the production of NO and PGE_2_. Chao et al. [27] considered that naringenin had a stronger inhibitory effect toward iNOS and COX-2 than did an equal concentration of vitamin C. Another study showed the possible interaction between naringenin and XO. Naoghare et al. [28] proposed that XO was also inhibited by naringenin because the concomitant hydrophilicity and hydrophobicity within the naringenin molecule helped it to bind the active site of XO more strongly, thereby reducing the activity of XO. In the present study, the pharmacological network built showed that naringenin interacted with iNOS and XO. In fact, COX-2 also exhibits interactions with naringenin, but as the rank was not in the top 10, the interaction was not shown in our network.

Although the working mechanism was described from molecular point of view, it had a strong theoretical and scientific implication. Partial predicted results have been reported in some literatures, serving as a good reference value for verifying our network. Moreover, we first reported that XO had interactions with eriocitrin, neoeriocitrin, isorhoifolin, and poncirin. The antioxidant activities of eriocitrin, neoeriocitrin, isorhoifolin, and poncirin and their inhibiting effects on oxidative or ROS damages had been investigated previously [29–32], but their inhibitory effects on XO were little reported. Hence, this screening method also provided important theoretical guidance for exploring XO inhibitors from TCM. Drug-target network indicated the molecular mechanism of action of ZZDHD with regard to antioxidant effects. In our future studies, the details and experimental verification for novel screened active compounds will be elucidated.

### 3.2. Antioxidative Molecular Mechanism of ZZDHD

The antioxidation mechanisms of ZZDHD were predicted using network pharmacology. Much research has revealed that CYP2E1 plays a role in ethanol-induced liver steatosis. Ethanol initially induces CYP2E1 activation followed by increases in oxidative stress in the liver [33], and the ROS generated by CYP2E1 promotes oxidative stress. ROS leads to the production of reactive aldehydes, also with potent proinflammatory properties [8]. Oxidative stress increases NF-*κ*B activation, thus enhancing the expression of COX-2 and iNOS [34]. The upregulation of COX-2 expression causes the enhanced production of PGE_2_, which functions through its receptors (EP_2_ and/or EP_4_) in hepatocytes to increase the accumulation of triglycerides [11], with further development in fatty liver. Recent studies have also shown that specific iNOS inhibitors or iNOS knockout protects against ethanol-caused oxidative stress [35, 36]. Other sources of prooxidant agent include acetaldehyde, an excellent substrate for the enzyme XO, which generates more toxic oxygen radicals during its oxidative catalysis [37]. Indeed, the inhibition of XO decreases ethanol- and acetaldehyde-induced lipid peroxidation.

Overproduction of ROS disrupts the balance between the oxidation and antioxidation systems, further causing oxidative stress [7]. Radical scavenging system is the body's first line of defense against oxidation, and SOD and GSH are the main components of the antioxidant protection system. SOD is a basic antioxidant enzyme responsible for catalyzing the dismutation of superoxide anion radical (O_2_^−•^), and its activity indirectly indicates the body's ability of scavenging free radical [38]. GSH is a major nonprotein thiol and plays a central role in coordinating the antioxidant defense process [39]. Depletion of GSH may be associated with aggravation of oxidative damage, which usually reflects the level of the organism's antioxidant ability. MDA, an end-product of lipid peroxidation, has been widely used as an indicator for the degree of lipid peroxidation [40] and it also indirectly incarnates the formation of free radicals and the degree of cell damage. Previous studies had reported that ZZDHD markedly improved SOD, GSH, and MDA, which indicated ZZDHD had contributed to the elimination of excessive oxygen free radicals and the reduction of lipid peroxidation [13].

**Figure 2:**
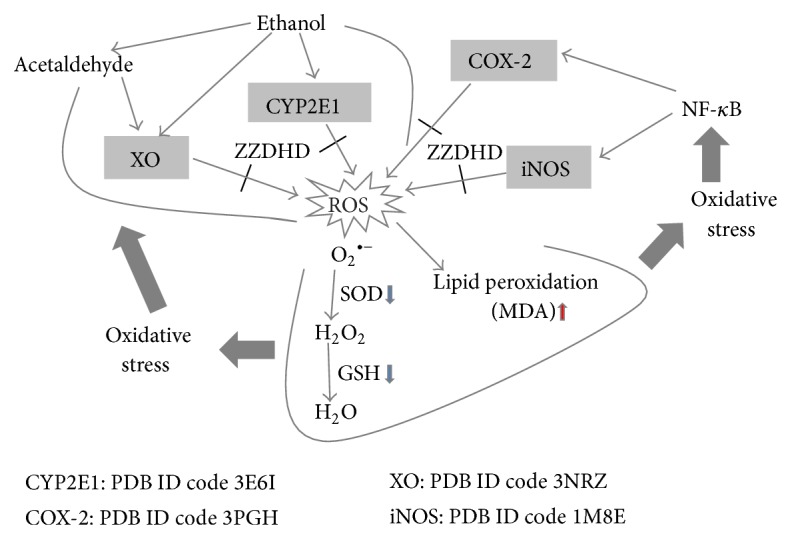
Schematic illustrating proposed action mechanism by which ZZDHD prevents ALD. That ethanol induces CPY2E1, and XO causes oxidative stress in liver, due to overproduction of ROS, antioxidants reduction, and lipid peroxidation. These results, in turn, improve NF-*κ*B activation and then increase the expression such as in COX-2 and iNOS, further aggravating oxidative injury. Multiple components from ZZDHD (e.g., aloe-emodin and naringenin) inhibit the activation of CYP2E1, XO, COX-2, and iNOS, thereby protecting liver against the damage.

ZZDHD modulated the activity of ethanol-metabolizing enzymes such as CYP2E1 and XO and inhibited the expression of enzymes such as COX-2 and iNOS. The above result showed that the antioxidant effect of ZZDHD may be an important influence factor for relieving oxidative stress as well as reducing the generation of lipid peroxidation induced by ethanol (Figure 2).

## 4. Conclusion

In this paper, a drug-target network was constructed through molecular docking and network analysis. The network predicted the underlying antioxidant mechanism of ZZDHD as an effective therapeutic approach to treat alcoholic liver disease. This study demonstrated that a network pharmacology-based approach was useful for elucidating the interrelationship between complex diseases such as ALD and TCM formula interventions. Therefore, network pharmacology is a forceful tool for exploring the potential mechanism of action of TCM formula and new active ingredients. As further steps, experimental verification of the potential effective compounds after candidate screening is needed to validate the interactions between drugs and proteins based on theoretical predictions.

## Supplementary Material

A total of thirty active components, including six iridoids, seventeen flavonoids, five anthraquinones and two coumarins were identified in ZZDHD using HPLC-PDA- ESI-MS/MS in our early laboratory work. These identified chemical structures were presented in Supplemental Fig.1 (see Figure S1), and all the structures of these compounds were optimized by MMFF94 Force Field in DS 2.5.
