# Supplementary material for: Network Pharmacology-Based Antioxidant Effect Study of Zhi-Zi-Da-Huang Decoction for Alcoholic Liver Disease
Source: Evid Based Complement Alternat Med. 2015 Apr 2;2015:492470. doi: 10.1155/2015/492470 (PMC4398926; doi:10.1155/2015/492470)
Supplement: Supplementary file 1 — A total of thirty active components, including six iridoids, seventeen flavonoids, five anthraquinones and two coumarins were identified in ZZDHD using HPLC-PDA- ESI-MS/MS in our early laboratory work. These identified chemical structures were presented in Supplemental Fig.1 (see Figure S1), and all the structures of these compounds were optimized by MMFF94 Force Field in DS 2.5. [file 492470.f1.pdf]

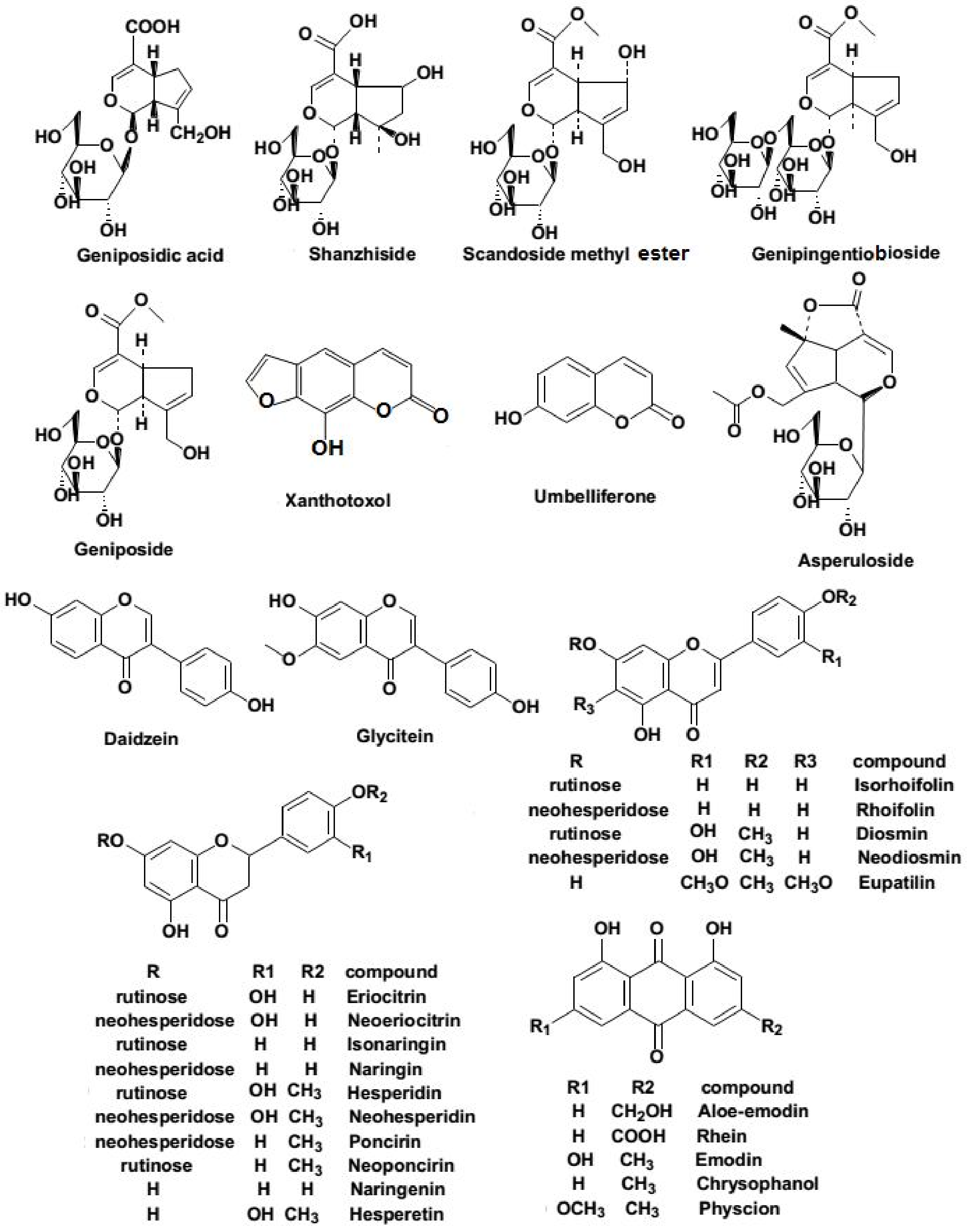

Figure S1 Chemical structures of the thirty identified components by HPLC-PDA- ESI-MS/MS in Zhi-Zi-Da-Huang Decoction.
